# The genome sequence of a ground beetle, *Harpalus rufipes *(DeGeer, 1774)

**DOI:** 10.12688/wellcomeopenres.22875.1

**Published:** 2024-08-14

**Authors:** Maxwell V. L. Barclay, Clive R. Turner, Natural History Museum Genome Acquisition Lab, Darwin Tree of Life Barcoding collective, Wellcome Sanger Institute Tree of Life Management, Samples and Laboratory team, Wellcome Sanger Institute Scientific Operations: Sequencing Operations, Wellcome Sanger Institute Tree of Life Core Informatics team, Tree of Life Core Informatics collective, Darwin Tree of Life Consortium

**Affiliations:** 1 Natural History Museum, London, England, UK

**Keywords:** Harpalus rufipes, ground beetle, genome sequence, chromosomal, Coleoptera

## Abstract

We present a genome assembly from an individual male *Harpalus rufipes* (a ground beetle; Arthropoda; Insecta; Coleoptera; Carabidae). The genome sequence spans 890.50 megabases. Most of the assembly is scaffolded into 19 chromosomal pseudomolecules, including the X sex chromosome. The mitochondrial genome has also been assembled and is 17.37 kilobases in length. Gene annotation of this assembly on Ensembl identified 13,884 protein-coding genes.

## Species taxonomy

Eukaryota; Opisthokonta; Metazoa; Eumetazoa; Bilateria; Protostomia; Ecdysozoa; Panarthropoda; Arthropoda; Mandibulata; Pancrustacea; Hexapoda; Insecta; Dicondylia; Pterygota; Neoptera; Endopterygota; Coleoptera; Adephaga; Caraboidea; Carabidae; Harpalinae; Harpalini; *Harpalus*; *Pseudoophonus*; *Harpalus (Pseudoophonus) rufipes* (DeGeer, 1774) (NCBI:txid247442).

## Background

The genome of a ground beetle, *Harpalus rufipes*, was sequenced as part of the Darwin Tree of Life Project, a collaborative effort to sequence all named eukaryotic species in the Atlantic Archipelago of Britain and Ireland. Here we present the first chromosomally complete genome sequence for *Harpalus rufipes*, based on a male specimen from Bookham Common, Surrey, UK.

*Harpalus rufipes* is a very common large ground beetle found in a wide range of habitats over most of the Palaearctic Region. Löbl & Löbl (2017) list records for more than 40 European countries, as well as most of North Africa and Central Asia, extending as far east as Xinjiang in China and East Siberia in Russia. It has also been introduced into North America where it is now common on the East Coast of the United States and Canada. It can be found across almost all of the British Isles, except for the north of Scotland. Adults can be very abundant especially on cultivated land, or ruderal places such as roadside verges and arable field margins ( Luff, 2007). They fly well and are strongly attracted to artificial lights on warm summer nights, sometimes in large numbers. Unlike most Carabidae, *Harpalus* species are not entirely predatory, but also feed on seeds. While they mostly consume seeds of ruderal plants, *H. rufipes* is sometimes considered a minor pest for eating the achenes (‘seeds’) from the outsides of strawberries (e.g. Lindroth, 1974), but an estimated 80% of the diet consists of invertebrates including several important pests ( Luff, 1980), making *H. rufipes* on balance a beneficial insect to agriculture.

*Harpalus rufipes* ranges in length from 11 to 16 mm, it is dull black with reddish legs and antennae, and its dorsal surface has conspicuous golden pubescence which immediately distinguishes it from most of its relatives. It is placed in the subgenus *Pseudoophonus* Motschulsky, sometimes treated as a full genus. Only two other *Pseudoophonus* species have been reported in Britain, *H. griseus* and *H. calceatus*, both of which are very rare.

A detailed study of the biology of *Harpalus rufipes* in Northumberland, England ( Luff, 1980) showed that adults were active from April to November, and then overwintered, with an overlapping new generation emerging in late summer. Adults are effective generalist pest controllers, because they are active early in the growing season and can reduce pest numbers while these are still low. Larvae live in the soil, usually feeding on weed and grass seeds, and can reach densities of 3 to 20 individuals per square metre.

## Genome sequence report

**Figure 1.**
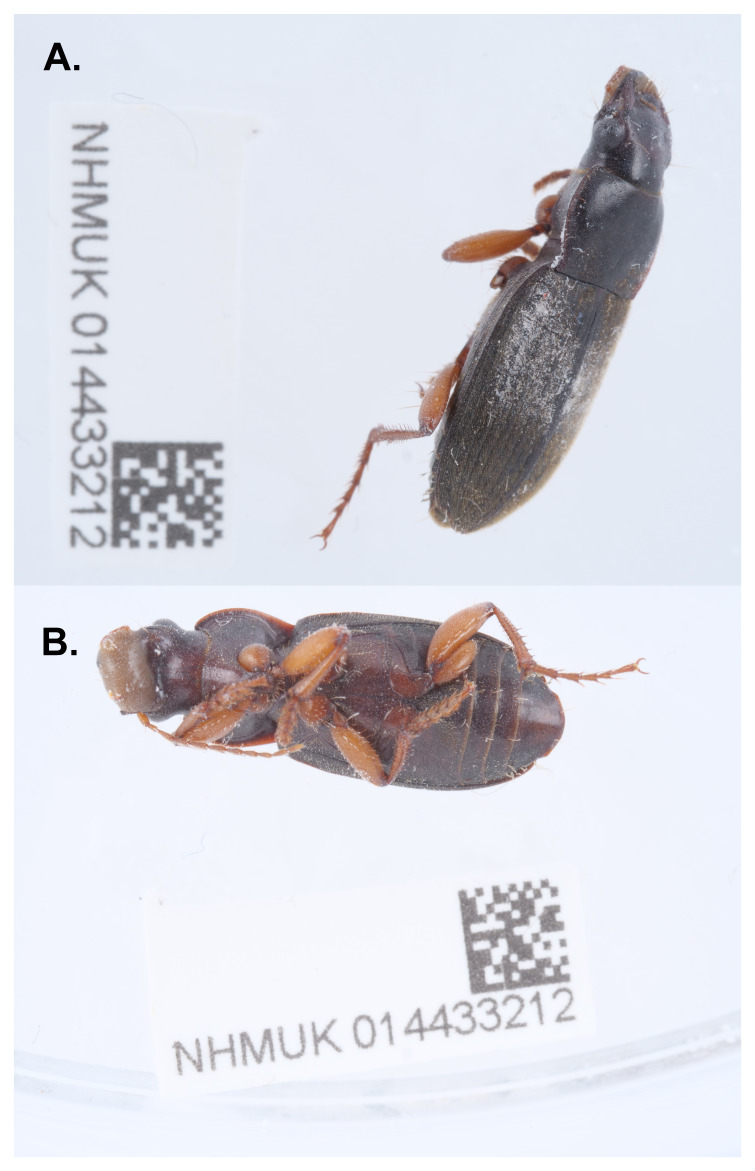
Photograph of the *Harpalus rufipes* (icHarRufp1) specimen used for genome sequencing.

The genome of an adult male *Harpalus rufipes* ( Figure 1) was sequenced using Pacific Biosciences single-molecule HiFi long reads, generating a total of 22.80 Gb (gigabases) from 2.28 million reads, providing approximately 43-fold coverage. Primary assembly contigs were scaffolded with chromosome conformation Hi-C data, which produced 114.43 Gbp from 757.81 million reads, yielding an approximate coverage of 129-fold. Specimen and sequencing information is summarised in Table 1.

**Table 1.** Specimen and sequencing data for *Harpalus rufipes*.

Project information			
**Study title**	Harpalus rufipes		
**Umbrella BioProject**	PRJEB59209		
**Species**	*Harpalus rufipes*		
**BioSample**	SAMEA9359449		
**NCBI taxonomy ID**	247442		
Specimen information			
**Technology**	**ToLID**	**BioSample accession**	**Organism part**
**PacBio long read sequencing**	icHarRufp1	SAMEA9359539	Abdomen
**Hi-C sequencing**	icHarRufp1	SAMEA9359538	Head and thorax
**RNA sequencing**	icHarRufp1	SAMEA9359539	Abdomen
Sequencing information			
**Platform**	**Run accession**	**Read count**	**Base count (Gb)**
**Hi-C Illumina NovaSeq 6000**	ERR10818311	7.58e+08	114.43
**PacBio Sequel IIe**	ERR10812854	1.84e+06	18.39
**PacBio Sequel IIe**	ERR10812855	2.28e+06	22.8
**RNA Illumina NovaSeq X**	ERR12765135	6.08e+07	9.18

**Figure 2.**
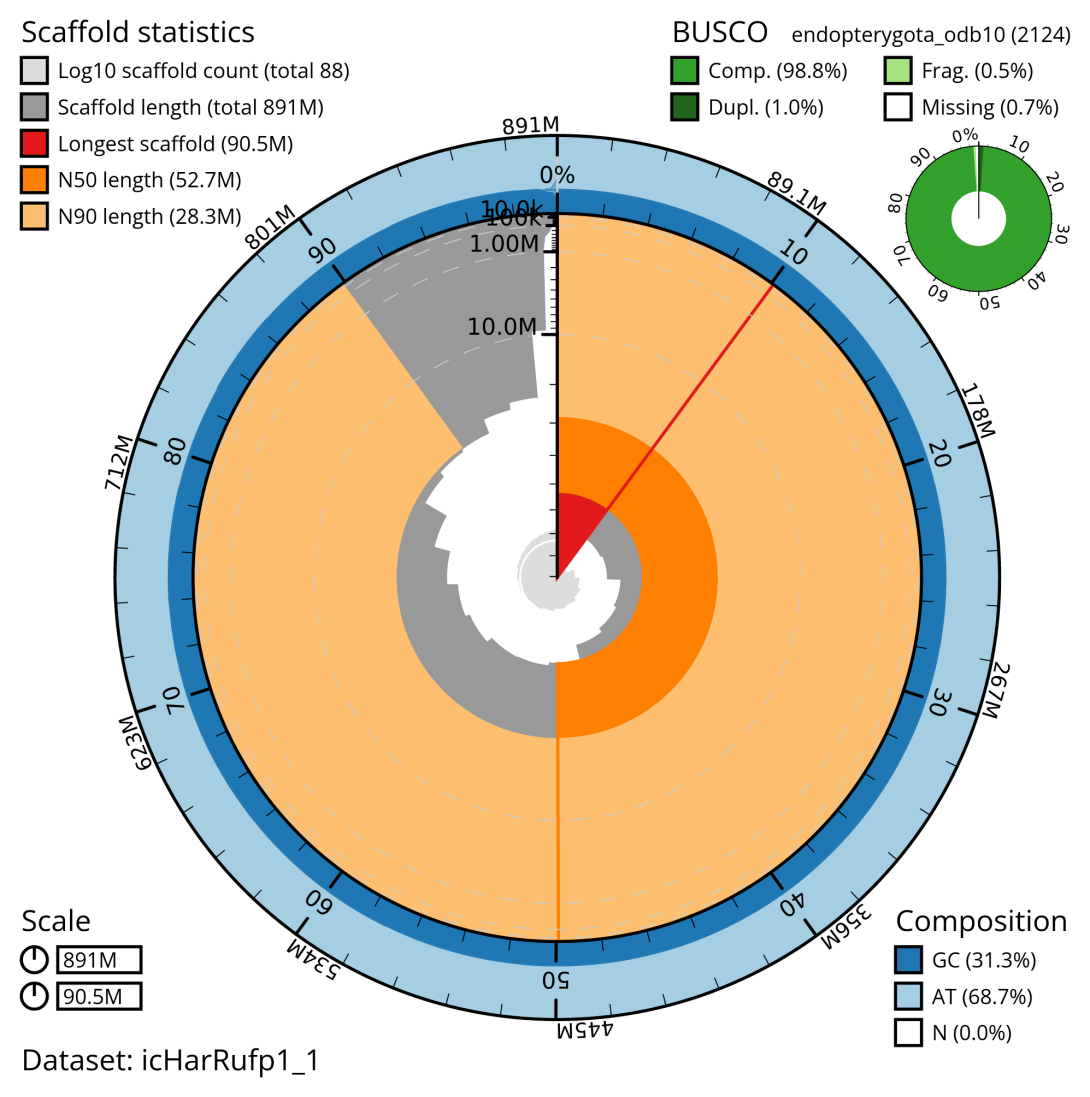
Genome assembly of *Harpalus rufipes*, icHarRufp1.1: metrics. The BlobToolKit snail plot shows N50 metrics and BUSCO gene completeness. The main plot is divided into 1,000 size-ordered bins around the circumference with each bin representing 0.1% of the 890,553,490 bp assembly. The distribution of scaffold lengths is shown in dark grey with the plot radius scaled to the longest scaffold present in the assembly (90,524,188 bp, shown in red). Orange and pale-orange arcs show the N50 and N90 scaffold lengths (52,722,989 and 28,314,201 bp), respectively. The pale grey spiral shows the cumulative scaffold count on a log scale with white scale lines showing successive orders of magnitude. The blue and pale-blue area around the outside of the plot shows the distribution of GC, AT and N percentages in the same bins as the inner plot. A summary of complete, fragmented, duplicated and missing BUSCO genes in the endopterygota_odb10 set is shown in the top right. An interactive version of this figure is available at https://blobtoolkit.genomehubs.org/view/icHarRufp1_1/dataset/icHarRufp1_1/snail.

**Figure 3.**
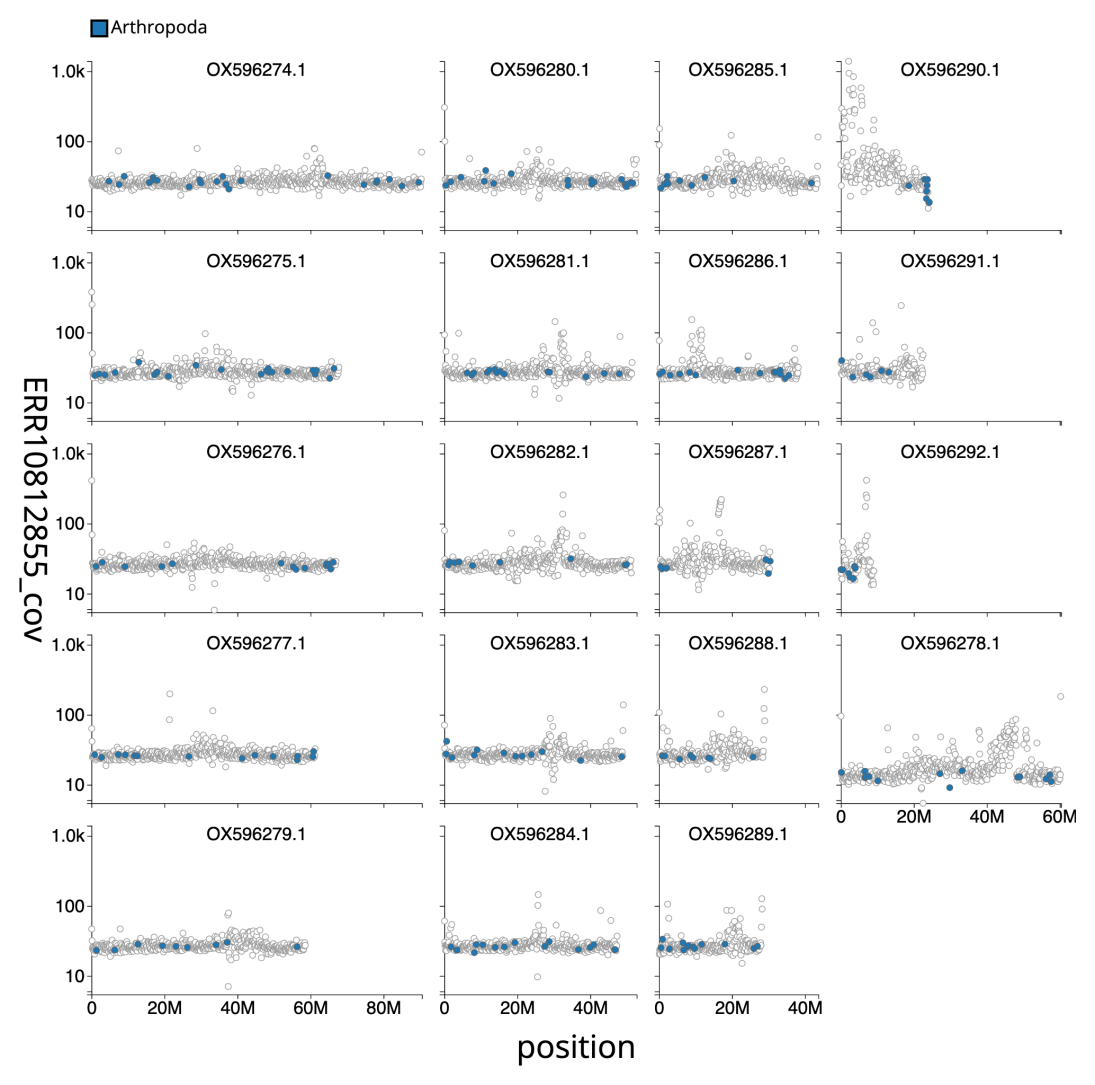
Genome assembly of *Harpalus rufipes*, icHarRufp1.1: Distribution plot of base coverage in ERR10812855 against position for sequences in the assembly. Windows of 100kb are coloured by phylum. The assembly has been filtered to exclude sequences with length < 2,550,000 An interactive version of this figure is available here.

**Figure 4.**
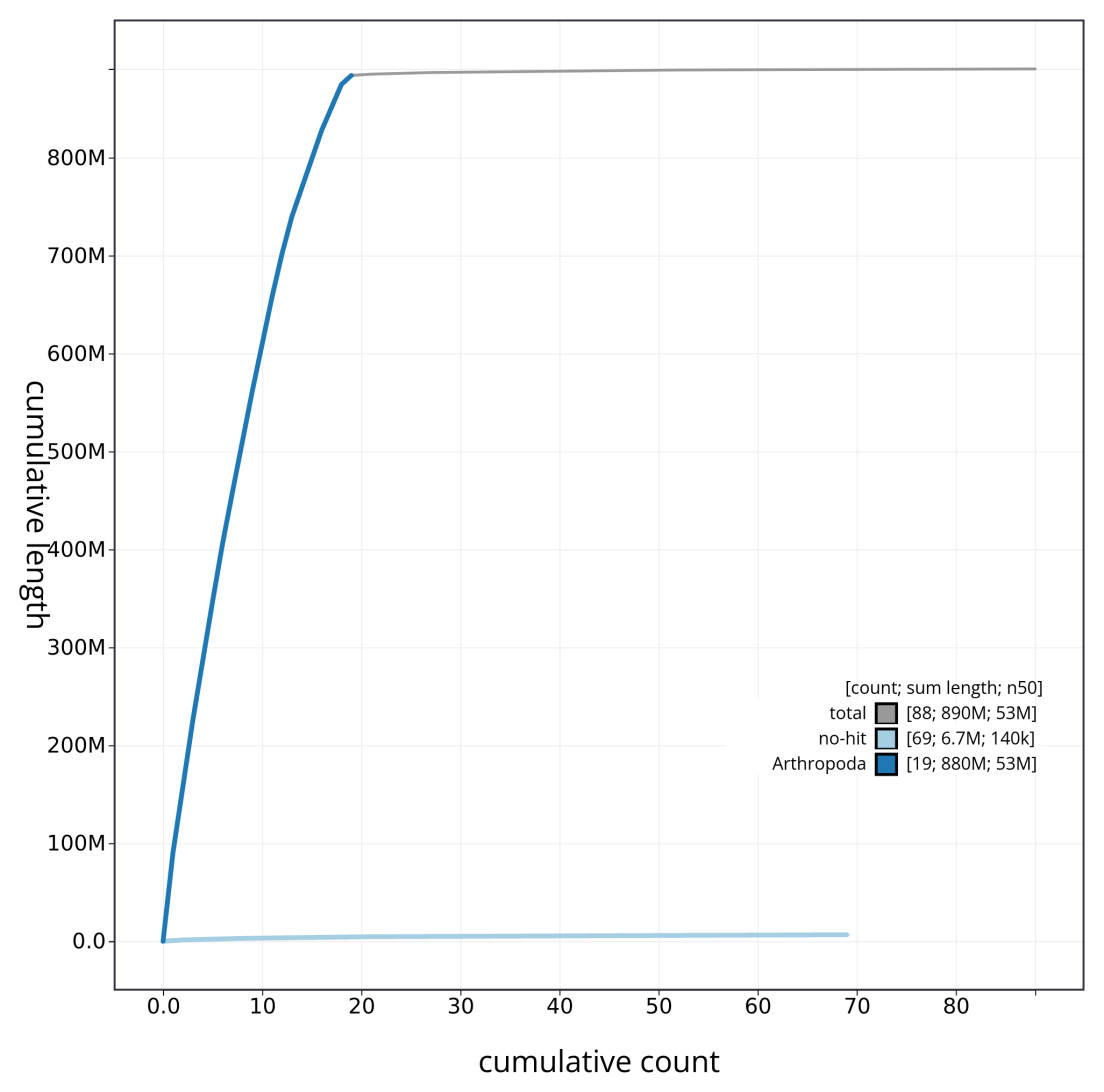
Genome assembly of *Harpalus rufipes* icHarRufp1.1: BlobToolKit cumulative sequence plot. The grey line shows cumulative length for all sequences. Coloured lines show cumulative lengths of sequences assigned to each phylum using the buscogenes taxrule. An interactive version of this figure is available at https://blobtoolkit.genomehubs.org/view/icHarRufp1_1/dataset/icHarRufp1_1/cumulative.

**Figure 5.**
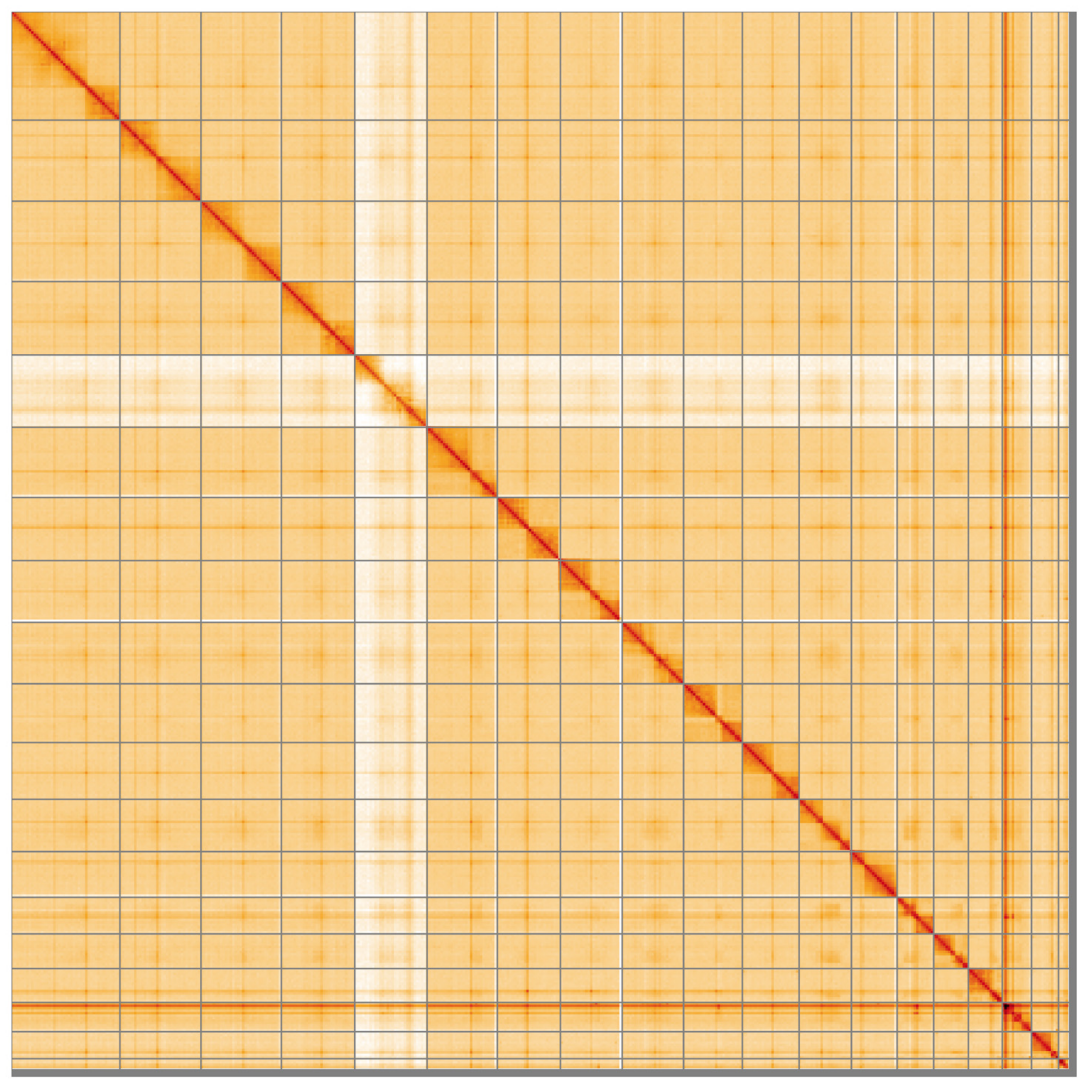
Genome assembly of *Harpalus rufipes* icHarRufp1.1: Hi-C contact map of the icHarRufp1.1 assembly, visualised using HiGlass. Chromosomes are shown in order of size from left to right and top to bottom. An interactive version of this figure may be viewed at https://genome-note-higlass.tol.sanger.ac.uk/l/?d=HwBjO53IRuCb4qV63cwCyg.

Manual assembly curation corrected 145 missing joins or mis-joins and 13 haplotypic duplications, reducing the assembly length by 0.81% and the scaffold number by 49.71%, and increasing the scaffold N50 by 97.76%. The final assembly has a total length of 890.50 Mb in 87 sequence scaffolds with a scaffold N50 of 52.7 Mb ( Table 2). The total count of gaps in the scaffolds is 262. The snail plot in Figure 2 provides a summary of the assembly statistics, while Figure 3 shows the distribution of base coverage against position for sequences in each chromosome of the assembly. The cumulative assembly plot in Figure 4 shows curves for subsets of scaffolds assigned to different phyla. Most (99.37%) of the assembly sequence was assigned to 19 chromosomal-level scaffolds, representing 18 autosomes and the X sex chromosome. Chromosome-scale scaffolds confirmed by the Hi-C data are named in order of size ( Figure 5; Table 3). While not fully phased, the assembly deposited is of one haplotype. Contigs corresponding to the second haplotype have also been deposited. The mitochondrial genome was also assembled and can be found as a contig within the multifasta file of the genome submission.

**Table 2.** Genome assembly data for *Harpalus rufipes*, icHarRufp1.1.

Genome assembly		
Assembly name	icHarRufp1.1	
Assembly accession	GCA_951394225.1	
*Accession of alternate haplotype*	*GCA_951394325.1*	
Span (Mb)	890.50	
Number of contigs	350	
Contig N50 length (Mb)	8.8	
Number of scaffolds	87	
Scaffold N50 length (Mb)	52.7	
Longest scaffold (Mb)	90.52	
Assembly metrics *		*Benchmark*
Consensus quality (QV)	57.8	*≥ 50*
*k*-mer completeness	99.99%	*≥ 95%*
BUSCO **	C:98.8%[S:97.8%,D:1.0%],F:0.5%,M:0.7%,n:2,124	*C ≥ 95%*
Percentage of assembly mapped to chromosomes	99.37%	*≥ 95%*
Sex chromosomes	X	*localised homologous pairs*
Organelles	Mitochondrial genome: 17.37 kb	*complete single alleles*
Genome annotation of assembly GCA_951394225.1 at Ensembl		
Number of protein-coding genes	13,884	
Number of non-coding genes	7,666	
Number of gene transcripts	30,774	

* Assembly metric benchmarks are adapted from column VGP-2020 of “Table 1: Proposed standards and metrics for defining genome assembly quality” from Rhie *et al.* (2021). ** BUSCO scores based on the endopterygota_odb10 BUSCO set using version 5.3.2. C = complete [S = single copy, D = duplicated], F = fragmented, M = missing, n = number of orthologues in comparison. A full set of BUSCO scores is available at https://blobtoolkit.genomehubs.org/view/icHarRufp1_1/dataset/icHarRufp1_1/busco.

**Table 3.** Chromosomal pseudomolecules in the genome assembly of *Harpalus rufipes*, icHarRufp1.

INSDC accession	Name	Length (Mb)	GC%
OX596274.1	1	90.52	31.0
OX596275.1	2	67.82	31.0
OX596276.1	3	67.09	31.0
OX596277.1	4	61.39	31.0
OX596279.1	5	58.66	31.5
OX596280.1	6	52.72	31.0
OX596281.1	7	51.6	31.0
OX596282.1	8	51.32	31.5
OX596283.1	9	49.1	31.5
OX596284.1	10	47.44	31.0
OX596285.1	11	43.67	31.5
OX596286.1	12	38.22	31.0
OX596287.1	13	30.55	32.0
OX596288.1	14	29.0	31.5
OX596289.1	15	28.31	31.5
OX596290.1	16	24.38	32.0
OX596291.1	17	22.63	32.0
OX596292.1	18	9.14	31.5
OX596278.1	X	60.34	31.0
OX596293.1	MT	0.02	19.5

The estimated Quality Value (QV) of the final assembly is 57.8 with *k*-mer completeness of 99.99%, and the assembly has a BUSCO v5.3.2 completeness of 98.8% (single = 97.8%, duplicated = 1.0%), using the endopterygota_odb10 reference set ( *n* = 2,124).

Metadata for specimens, BOLD barcode results, spectra estimates, sequencing runs, contaminants and pre-curation assembly statistics are given at https://links.tol.sanger.ac.uk/species/247442.

## Genome annotation report

The *Harpalus rufipes* genome assembly (GCA_951394225.1) was annotated at the European Bioinformatics Institute (EBI) on Ensembl Rapid Release. The resulting annotation includes 30,774 transcribed mRNAs from 13,884 protein-coding and 7,666 non-coding genes ( Table 2; https://rapid.ensembl.org/Harpalus_rufipes_GCA_951394225.1/Info/Index). The average transcript length is 15,373.62. There are 1.43 coding transcripts per gene and 4.52 exons per transcript.

## Methods

### Sample acquisition

An adult male *Harpalus rufipes* (specimen ID NHMUK014433212, ToLID icHarRufp1) was collected from Bookham Common, Leatherhead, England, UK on 2021-04-18. The specimen was collected and identified by Maxwell Barclay (Natural History Museum) and preserved by dry freezing at –80 °C.

The initial species identification was verified by an additional DNA barcoding process according to the framework developed by Twyford *et al*. (2024). A small sample was dissected from the specimen and stored in ethanol, while the remaining parts of the specimen were shipped on dry ice to the Wellcome Sanger Institute (WSI). The tissue was lysed, the COI marker region was amplified by PCR, and amplicons were sequenced and compared to the BOLD database, confirming the species identification ( Crowley *et al.*, 2023). Following whole genome sequence generation, the relevant DNA barcode region was also used alongside the initial barcoding data for sample tracking at the WSI ( Twyford *et al.*, 2024). The standard operating procedures for Darwin Tree of Life barcoding have been deposited on protocols.io ( Beasley *et al.*, 2023).

### Nucleic acid extraction

The workflow for high molecular weight (HMW) DNA extraction at the WSI Tree of Life Core Laboratory includes a sequence of core procedures: sample preparation and homogenisation, DNA extraction, fragmentation, and DNA purification. Protocols developed by the WSI Tree of Life Core Laboratory are publicly available on protocols.io ( Denton *et al.*, 2023b).

The icHarRufp1 sample was weighed and dissected on dry ice ( Jay *et al.*, 2023). Tissue from abdomen was homogenised using a PowerMasher II tissue disruptor ( Denton *et al.*, 2023a). HMW DNA was extracted using the Automated MagAttract v1 protocol ( Sheerin *et al.*, 2023). DNA was sheared into an average fragment size of 12–20 kb in a Megaruptor 3 system with speed setting 30 ( Todorovic *et al.*, 2023). Sheared DNA was purified by solid-phase reversible immobilisation, using AMPure PB beads to sample to eliminate shorter fragments and concentrate the DNA ( Strickland *et al.*, 2023). The concentration of the sheared and purified DNA was assessed using a Nanodrop spectrophotometer and Qubit Fluorometer using the Qubit dsDNA High Sensitivity Assay kit. Fragment size distribution was evaluated by running the sample on the FemtoPulse system.

RNA was extracted from abdomen tissue of icHarRufp1 in the Tree of Life Laboratory at the WSI using the RNA Extraction: Automated MagMax™ *mir*Vana protocol ( do Amaral *et al.*, 2023). The RNA concentration was assessed using a Nanodrop spectrophotometer and a Qubit Fluorometer using the Qubit RNA Broad-Range Assay kit. Analysis of the integrity of the RNA was done using the Agilent RNA 6000 Pico Kit and Eukaryotic Total RNA assay.

### Sequencing

Pacific Biosciences HiFi circular consensus DNA sequencing libraries were constructed according to the manufacturers’ instructions. Poly(A) RNA-Seq libraries were constructed using the NEB Ultra II RNA Library Prep kit. DNA and RNA sequencing was performed by the Scientific Operations core at the WSI on Pacific Biosciences Sequel IIe (HiFi) and Illumina NovaSeq X (RNA-Seq) instruments. Hi-C data were also generated from head and thorax tissue of icHarRufp1 using the Arima-HiC v2 kit. The Hi-C sequencing was performed using paired-end sequencing with a read length of 150 bp on the Illumina NovaSeq 6000 instrument.

### Genome assembly, curation and evaluation

***Assembly***

Original assembly of HiFi reads was performed using Hifiasm ( Cheng *et al.*, 2021) with the --primary option. Haplotypic duplications were identified and removed with purge_dups ( Guan *et al.*, 2020). Hi-C reads were mapped with bwa-mem2 ( Vasimuddin *et al.*, 2019) to the primary contigs, which were further scaffolded using the provided Hi-C data ( Rao *et al.*, 2014) in YaHS ( Zhou *et al.*, 2023) using the --break option. Scaffolded assemblies were evaluated using Gfastats ( Formenti *et al.*, 2022), BUSCO ( Manni *et al.*, 2021) and MERQURY.FK ( Rhie *et al.*, 2020).

The mitochondrial genome was assembled using MitoHiFi ( Uliano-Silva *et al.*, 2023), which runs MitoFinder ( Allio *et al.*, 2020) and uses these annotations to select the final mitochondrial contig and to ensure the general quality of the sequence.

***Assembly curation***

The assembly was decontaminated using the Assembly Screen for Cobionts and Contaminants (ASCC) pipeline (article in preparation). Manual curation was primarily conducted using PretextView ( Harry, 2022), with additional insights provided by JBrowse2 ( Diesh *et al.*, 2023) and HiGlass ( Kerpedjiev *et al.*, 2018). Scaffolds were visually inspected and corrected as described by Howe *et al*. (2021). Any identified contamination, missed joins, and mis-joins were corrected, and duplicate sequences were tagged and removed. The sex chromosome was identified by read coverage statistics. The entire process is documented at https://gitlab.com/wtsi-grit/rapid-curation (article in preparation).

***Evaluation of the final assembly***

A Hi-C map for the final assembly was produced using bwa-mem2 ( Vasimuddin *et al.*, 2019) in the Cooler file format ( Abdennur & Mirny, 2020). To assess the assembly metrics, the *k*-mer completeness and QV consensus quality values were calculated in Merqury ( Rhie *et al.*, 2020). This work was done using Nextflow ( Di Tommaso *et al.*, 2017) DSL2 pipelines “sanger-tol/readmapping” ( Surana *et al.*, 2023a) and “sanger-tol/genomenote” ( Surana *et al.*, 2023b). The genome was analysed within the BlobToolKit environment ( Challis *et al.*, 2020) and BUSCO scores ( Manni *et al.*, 2021; Simão *et al.*, 2015) were calculated.

The genome evaluation pipelines were developed using the nf-core tooling ( Ewels *et al.*, 2020), use MultiQC ( Ewels *et al.*, 2016), and make extensive use of the Conda package manager, the Bioconda initiative ( Grüning *et al.*, 2018), the Biocontainers infrastructure ( da Veiga Leprevost *et al.*, 2017), and the Docker ( Merkel, 2014) and Singularity ( Kurtzer *et al.*, 2017) containerisation solutions.

Table 4 contains a list of relevant software tool versions and sources.

**Table 4.** Software tools: versions and sources.

Software tool	Version	Source
BlobToolKit	4.2.1	https://github.com/blobtoolkit/blobtoolkit
BUSCO	5.3.2	https://gitlab.com/ezlab/busco
Hifiasm	0.16.1-r375	https://github.com/chhylp123/hifiasm
HiGlass	1.11.6	https://github.com/higlass/higlass
Merqury	MerquryFK	https://github.com/thegenemyers/MERQURY.FK
MitoHiFi	2	https://github.com/marcelauliano/MitoHiFi
PretextView	0.2	https://github.com/wtsi-hpag/PretextView
purge_dups	1.2.3	https://github.com/dfguan/purge_dups
sanger-tol/genomenote	v1.0	https://github.com/sanger-tol/genomenote
sanger-tol/readmapping	1.1.0	https://github.com/sanger-tol/readmapping/tree/1.1.0
YaHS	yahs-1.1.91eebc2	https://github.com/c-zhou/yahs

### Genome annotation

The Ensembl Genebuild annotation system ( Aken *et al.*, 2016) was used to generate annotation for the *Harpalus rufipes* assembly (GCA_951394225.1) in Ensembl Rapid Release at the EBI. Annotation was created primarily through alignment of transcriptomic data to the genome, with gap filling via protein-to-genome alignments of a select set of proteins from UniProt ( UniProt Consortium, 2019).

### Wellcome Sanger Institute – Legal and Governance

The materials that have contributed to this genome note have been supplied by a Darwin Tree of Life Partner. The submission of materials by a Darwin Tree of Life Partner is subject to the **‘Darwin Tree of Life Project Sampling Code of Practice’**, which can be found in full on the Darwin Tree of Life website here. By agreeing with and signing up to the Sampling Code of Practice, the Darwin Tree of Life Partner agrees they will meet the legal and ethical requirements and standards set out within this document in respect of all samples acquired for, and supplied to, the Darwin Tree of Life Project.

Further, the Wellcome Sanger Institute employs a process whereby due diligence is carried out proportionate to the nature of the materials themselves, and the circumstances under which they have been/are to be collected and provided for use. The purpose of this is to address and mitigate any potential legal and/or ethical implications of receipt and use of the materials as part of the research project, and to ensure that in doing so we align with best practice wherever possible. The overarching areas of consideration are:

•   Ethical review of provenance and sourcing of the material

•   Legality of collection, transfer and use (national and international)

Each transfer of samples is further undertaken according to a Research Collaboration Agreement or Material Transfer Agreement entered into by the Darwin Tree of Life Partner, Genome Research Limited (operating as the Wellcome Sanger Institute), and in some circumstances other Darwin Tree of Life collaborators.

## Data Availability

European Nucleotide Archive: Harpalus rufipes. Accession number PRJEB59209; https://identifiers.org/ena.embl/PRJEB59209 ( Wellcome Sanger Institute, 2023). The genome sequence is released openly for reuse. The *Harpalus rufipes* genome sequencing initiative is part of the Darwin Tree of Life (DToL) project. All raw sequence data and the assembly have been deposited in INSDC databases. Raw data and assembly accession identifiers are reported in Table 1 and Table 2.

## Author information

Members of the Natural History Museum Genome Acquisition Lab are listed here: https://doi.org/10.5281/zenodo.12159242.

Members of the Darwin Tree of Life Barcoding collective are listed here: https://doi.org/10.5281/zenodo.12158331

Members of the Wellcome Sanger Institute Tree of Life Management, Samples and Laboratory team are listed here: https://doi.org/10.5281/zenodo.12162482.

Members of Wellcome Sanger Institute Scientific Operations: Sequencing Operations are listed here: https://doi.org/10.5281/zenodo.12165051.

Members of the Wellcome Sanger Institute Tree of Life Core Informatics team are listed here: https://doi.org/10.5281/zenodo.12160324.

Members of the Tree of Life Core Informatics collective are listed here: https://doi.org/10.5281/zenodo.5013541.

Members of the Darwin Tree of Life Consortium are listed here: https://doi.org/10.5281/zenodo.4783558.
