# Supplementary material for: The genome sequence of a ground beetle, Harpalus rufipes (DeGeer, 1774)
Source: Wellcome Open Res. 2024 Aug 14;9:483. [Version 1] doi: 10.12688/wellcomeopenres.22875.1 (PMC12375188; doi:10.12688/wellcomeopenres.22875.1)
Supplement: Peer review history

# Reviewer report: Reviewer response for version 1

**DOI:** 10.21956/wellcomeopenres.25190.r126346

**Published:** 2025-08-23

**Authors:** Henrique Antoniolli

**Affiliations:** 1 Universidade Federal do Rio Grande do Sul (UFRGS), Porto Alegre, Brazil

The present study reports a highly contiguous genome assembly for a ground beetle, *Harpalus rufipes*. Methods and protocols are well described, following established procedures for the Darwin Tree of Life project. The resulting nuclear genome shows satisfactory statistics of completeness and annotation. However, I recommend the authors to also deposit the mitochondrial genome with full gene annotations.

Are sufficient details of methods and materials provided to allow replication by others?

Yes

Is the rationale for creating the dataset(s) clearly described?

Yes

Are the datasets clearly presented in a useable and accessible format?

Yes

Are the protocols appropriate and is the work technically sound?

Yes

Reviewer Expertise:

Genomics, Evolution

I confirm that I have read this submission and believe that I have an appropriate level of expertise to confirm that it is of an acceptable scientific standard.

# Reviewer report: Reviewer response for version 1

**DOI:** 10.21956/wellcomeopenres.25190.r97017

**Published:** 2024-09-24

**Authors:** Ranjit Kumar Sahoo

**Affiliations:** 1 CSIR-Centre for Cellular and Molecular Biology (CCMB), Hyderabad, Telengana, India

The data note by Barclay and Turner presents the first genome assembly, of chromosome level, of a ground beetle *Harpalus rufipes*, which belongs to one of the species-rich beetle families, Carabidae. The current study is part of the collaborative effort from the Darwin Tree of Life Project which aims to assemble genomes of known eukaryotes from the Atlantic Archipelago of Britain and Ireland. Using the abdominal tissue of one adult male specimen from Bookham Common, England, the authors gathered 22.8 Gb whole genome and 9.18 Gb transcriptome sequence data for the beetle. They applied updated analytical tools to assemble and annotate the genome. This resulted in a primary genome assembly in 350 contigs with the N50 value of 8.8 Mb. Additionally, DNA from head and thorax tissues were sequenced for conformation data (114.43 Gb) further to scaffold the assembly into 87 scaffolds with the N50 improved to 52.7 Mb. Following contamination and duplication check, and manual curation to correct any missed joins, ca. 99.37% of the scaffolds were assigned to 18 autosomes and one sex-chromosome. The resulting final assembly, of 890 Mb, was finally evaluated for k-mer completeness (99.99%), QV consensus quality (57.8%), and BUSCO completeness (98.8%). The genome assembly was finally annotated using Ensembl Genebuild to find 30774 transcripts from 13884 protein-coding and 7666 non-coding genes. Additionally, the complete mitochondrial genome of 17.37 Kb was assembled.

 The study follows standardized methods for genome assembly, as adapted by the Darwin Tree of Life Project, to generate data that has definite academic merit and is useful for subsequent comparative or meta-analysis.

 Moreover, I have mentioned few comments below for consideration.

**Background:**

 Löbl & Löbl (2017) records the presence of this species from more than 40 European countries….

 …making *H. rufipes* effectively a beneficial insect to agriculture.

 The last part of the paragraph 2 (starting from ‘Unlike most Carabidae…’) can suitably be combined with the paragraph 4 to provide a comprehensive overview to the potential effect of the beetle on the pest control.

**Genome sequence report:**

- Karyotype: Authors assigned most of the scaffolds to 19 chromosome pseudomolecules. At this stage, it’s relevant to reflect on the karyotype of the species or genus if it’s been reported in the literature. Such reflection further confirms the validity of the higher-level assembly. For instance, Serrano and Yadav (1984) presents the karyotype formulae for a large number of Adephaga beetles revealing the common occurrence of both XO (male): XX (female) and XY: XX types of sex determination. This article further compiles the formulae for many species of *Harpalus*, to which the current study species belongs, showing that 2n+X is the common male type in this genus. Authors may further refer to any other investigations on *Harpalus rufipes* karyotype if it’s reported.
- Figure 3: I find this figure unintuitive and difficult to interpret. Particularly, I couldn’t make sense of the colored dots in the graph. Authors may describe them clearly in the figure title and if required, mention their relevance in the main text.
- Figure 5: To make this figure more informative, I suggest adding the chromosome name or accessions (as per Table 3) to the X or Y axis, if applicable. This would help the readers to quickly infer any patterns observed in the graph, for instance, the distinct color pattern in the contact map of the fifth chromosome may correspond to the sex-chromosome.
- Mitogenome: I think it’s relevant to mention the size of the mitochondrial genome in the main text. It’s equally important to contrast the mitochondrial genome against the whole genome: whether 890.50 Mb includes both nuclear and mitochondrial genomes. For instance, I would prefer to rewrite the sentence as, “The mitochondrial genome of 17.37 Kb was assembled out of 890.50 Mb total genome size, and it can be found as a contig (provide fasta identifier) within the multifasta file of the genome submission”. Contig identifier can also be added to the organelles assembly matrices segment in Table 2.

Are sufficient details of methods and materials provided to allow replication by others?

Yes

Is the rationale for creating the dataset(s) clearly described?

Yes

Are the datasets clearly presented in a useable and accessible format?

Yes

Are the protocols appropriate and is the work technically sound?

Yes

Reviewer Expertise:

Population genomics, Wolbachia biology, Trait evolution

I confirm that I have read this submission and believe that I have an appropriate level of expertise to confirm that it is of an acceptable scientific standard.

## References

[1] Chromosome Numbers and Sex-Determining Mechanisms in Adephagan Coleoptera. *The Coleopterists Bulletin JSTOR* .1984,vol. 38, no. 4, pp. 335–57; Reference source
